# Prioritized factors of severity prognosis in patients admitted for sepsis or septic shock in a resource-limited region of central Vietnam

**DOI:** 10.62838/jccm-2026-0033

**Published:** 2026-07-27

**Authors:** Thinh Tran Xuan, Lien Truong Thuc, Minh Nguyen Van, Cao Khoa Dang, Nhan Phuc Thanh Nguyen, Thong Tran Huu, Nancy Ha, Thang Phan

**Affiliations:** Hue University of Medicine and Pharmacy, Hue University,Vietnam; Department of Anesthesiology, Vinmec Nha Trang International Hospital; PhD student, Faculty of Public Health, Thammasat University, Rangsit campus,Thailand; Center for Emergency Medicine, Bach Mai Hospital, Hanoi, Vietnam; Stanford University,United States

**Keywords:** Sepsis, septic shock, lactate/albumin, platelets, severity prognosis

## Abstract

**Objectives:**

The early diagnosis and prognostication of severity in sepsis plays a crucial role in determining appropriate treatment strategies. This study aimed to assess the role of cheaper prognostic factors including lactate/albumin ratio and platelet count in patients with sepsis as they are correlated with the rate of multiorgan failure, and mortality rate.

**Methods:**

A cross-sectional study was conducted on 146 adult patients diagnosed with sepsis and septic shock according to the Sepsis-3 definition. All patients were admitted at either the intensive care unit of Hue University of Medicine and Pharmacy Hospital or Hue Central Hospital.

**Results:**

Average age of patients was 67 years-old, with 37.7% having sepsis and 62.3% with septic shock. The overall mortality rate in the study was 51.4% (75/146 patients). Forty six patients (32.5%) had thrombocytopenia, with mild, moderate, and severe decreases accounting for 8.9%, 21.2%, and 1.4%, respectively. The lactate/albumin ratio with a cutoff of 2.1 was the best prognostic factor for mortality with an AUROC of 0.76, sensitivity of 58.7%, and specificity of 88.7% compared to other biomarkers including lactate (AUROC = 0.71), albumin (AUROC = 0.74), platelet count (AUROC = 0.65), white blood cell count (AUROC = 0.48), procalcitonin (AUROC = 0.43), and BE (AUROC = 0.44). Multivariate regression analysis identified the L/A ratio, platelet count, SOFA score as significant predictors of mortality in sepsis.

**Conclusion:**

The lactate/albumin ratio with a cutoff of 2.1 is an available effective tool for severity prognosis of patients with sepsis/septic shock in developing countries.

## Introduction

Certain physiologic processes associated with sepsis and septic shock are the body's natural response to the invasion by pathogenic bacteria and their toxins. However, these processes often lead to hemodynamic instability and multiorgan failure as a result of decreased perfusion and reduced oxygen supply to tissues. Despite advancements in early diagnosis and treatment, sepsis and septic shock remain a significant global health challenge, especially in developing countries where the mortality rate can be as high as 50% [1]. Early diagnosis and prognostication of severity plays a crucial role in determining treatment strategies aimed at reducing mortality rates, decreasing length of hospital stays, and lowering treatment costs. Various scoring systems and markers are used in clinical practice for prognostication of disease severity such as the SOFA score, APACHE II, procalcitonin, pre-pepsin, IL8, TNF-α, CRP, lactate, BE, albumin [2]. Lactate and albumin are two independent prognostic indicators of mortality in sepsis and septic shock [3]. However, these values can be confounded by various other factors such as pre-existing organ failure, diabetes, and/or medications. The combination of the lactate/albumin ratio (L/A) may be more helpful in enhancing the reliability of mortality prognosis in critically ill patients recently [4]. In addition, platelet count may also serve as a surrogate for disease severity. In the context of sepsis, the quantity, ratio, and function of various blood cells often undergo significant changes, especially platelets. Decreased platelets may be observed in patients admitted to the intensive care unit with sepsis at an incidence of around 40% [5, 6]. Decreased platelets and increased mean platelet volume are considered manifestations of hematopoietic dysfunction, coagulation disorders, and are associated with prolonged hospital stays and increased mortality rates in sepsis patients [7, 8]. Given that there are multiple methods of predicting disease severity in sepsis, this study aims to evaluate the prognostic capabilities of several different available bio-markers in a resource-limited setting including lactate/albumin ratio, lactate, albumin, procalcitonin as well as platelet count.

## Methods

### 2.1. Inclusion criteria

Patients over 18 years old who were admitted to either the intensive care unit of Hue University of Medicine and Pharmacy Hospital or Hue Central Hospital and were diagnosed with sepsis or septic shock according to the definition and criteria established by the Third International Consensus for Sepsis and Septic Shock (Sepsis-3) [9].

– Sepsis: Determined when there is an infection and multiorgan dysfunction (acute change in the SOFA score ≥ 2 points).
– Septic shock: Defined as a patient with sepsis who has been adequately fluid resuscitated, but (1) requires vasopressors to maintain a mean blood pressure ≥ 65 mmHg and (2) serum lactate level > 2 mmol/L.

### 2.2. Exclusion criteria

– Refusal to participate in the study
– Nosocomial infections
– Pre-existing severe disease including acute leukemia, end-stage cancer, AIDS, decompensated cirrhosis, and immune thrombocytopenia
– Albumin or platelets transfusion prior to admission to the intensive care unit.

### 2.3. Methodology

#### 2.3.1. Study Design: Cross-sectional study

#### 2.3.2. Sample size:

The sample size was determined using the estimation method for ROC curve analysis described by Hanley and McNeil [10] and implemented with MedCalc software. Previous evidence from Phan Kim Chau Man et al. reported an area under the curve (AUC) of 0.866 for lactate concentration in predicting severe outcomes among patients with sepsis [11]. In the present study, the null hypothesis (H_0_) assumed an AUC of 0.70, with a two-sided significance level of 0.05 and a statistical power (1 − β) of 90%. A negative-to-positive group ratio of 2:1 was specified. Under these assumptions, the minimum required sample size was 102 patients, comprising 34 patients in the positive group and 68 in the negative group. After inflating the sample size by 20% to account for potential dropout or incomplete data, the required sample size was estimated at 125 patients. In fact, a total of 146 patients were ultimately enrolled in the study.

#### 2.3.3. Study period and location

– Study period: From February 2022 to May 2023.
– Study location: Department of Anesthesiology, Emergency and critical Care, Hue University of Medicine and Pharmacy Hospital and Intensive care unit of Hue Central Hospital.
– Duration of intensive care treatment: Time from admission to intensive care until discharge.
– Mortality rate: Includes patients who died from sepsis and septic shock in the intensive care unit, excluding patients who died from hospital-acquired infections.

#### 2.3.4. Data collection

Baseline data of patients, including gender, age, vital signs, comorbidities, clinical and laboratory characteristics were collected. Venous blood samples were collected from patients immediately after admission to the intensive care unit.

#### 2.3.5. Statistical analysis

SPSS 20.0 statistical and MedCalc 22.017 software was used for data analysis. Enumeration data were presented as n (%) and the χ^2^ test was used for comparison between groups. U test was used for ranked data. Measurement data with a normal distribution were presented as X±sd, and t-test was used for comparison between groups. Those with a skewed distribution were presented as the median (P25, P75), and the Mann-Whitney U test was used for comparison between groups and Kruskal Wallis H test for comparison among multiple groups. The predictive value of serum lactate, albumin, L/A, procalcitonin, BE, white blood cells count for severity prognosis in patients with sepsis was analyzed using ROC curves. Delong test was used for comparing ROC values. A multivariate logistic regression model was used to identify independent prognostic factors for mortality among sepsis patients. Differences with a p value of <0.05 were considered statistically significant.

#### 2.3.6. Study flowchart

**Figure:**
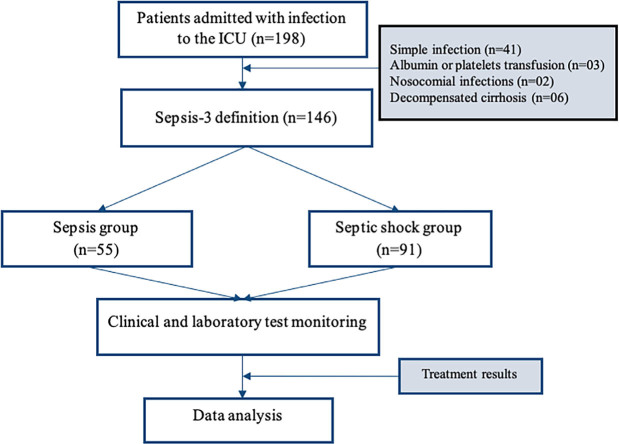


## Results

Data from a total of 146 patients diagnosed with sepsis or septic shock according to Sepsis-3 criteria from February 2022 to May 2023 were obtained.

**Table 1:** Socio-demographic variables of TBI patients

**Age (years), Median (IQR)**	**67 (29–107)**	
Gender (%)	Male	89 (61%)
	Female	57 (39%)
Platelet count	Sepsis group	55 (37.7%)
	Septic shock group	91 (62.3%)
	Normal (≥150 G/L)	100 (68.5%)
	Low (100 – 149 G/L)	13 (8.9%)
	Intermediate (50 – 99 G/L)	31 (21.2%)
	Very low (<50 G/L)	2 (1.4%)
Infection source	Gastrointestinal tract	64 (43.8%)
	Respiratory tract	50 (34.2%)
	Urinary tract	20 (13.7%)
	Skin	9 (6.2%)
	Unknown	3 (2.1%)
Bacteria isolated in blood (n = 37)	K. pneumoniae	11 (29.7%)
	P. aeruginosa	4 (10.8%)
	S. aureus	4 (10.8%)
	E.coli	9 (24.3%)
	A. baumannii	1 (2.7%)
	Other	11 (29.7%)

### 3.1. General characteristics

The proportion of patients diagnosed with sepsis and septic shock in the study was 37.7% and 62.3%, respectively. Forty-six patients (32.5%) had decreased platelets. Abdominal infections were the most common focus of infection sources (43.8%), followed by respiratory infections (34.2%). The most common pathogens isolated in blood were K. pneumoniae (29.7%) and E. coli (24.3%).

**Table 2:** Clinical and laboratory characteristics

**Group Variables**	**Total (n = 146)**	**Sepsis (n = 55)**	**Septic shock (n = 91)**	**p**	**Normal platelet (n = 100)**	**Decreased platelets (n = 46)**	**p**
HR (beats/min)	105.5 ± 21.7	102.3 ± 17.9	107.4 ± 23.7	>0.05	105.6 ± 21.9	105.2 ± 21.6	
RR (beats/min)	22.1 ± 6.2	23.9 ± 5.2	21.1 ± 6.5		22.7 ± 6.1	20.7 ± 6	
MBP (mmHg)	78.1 ± 20.1	84.5 ± 15.5	74.3 ± 21.6	<0.05	79.2 ± 20.6	75.7 ± 19.7	>0.05^(a)^
SOFA score	7 (6.1 – 7.2)	4 (4.0 – 5.3)	8 (7.3 – 8.5)	<0.05	5 (4.9 – 6.0)	9 (8.4 – 9.9)	<0.05^(b)^
WBC (G/L)	14.3 ± 10	16.3 ± 10	13.1 ± 9.9	>0.05	15.6 ± 9.5	11.4 ± 10.6	
PLT (G/L)	197.5 ± 100.5	228.8 ± 100.3	178.5 ± 96.2		246.6 ± 82.6	90.5 ± 20.8	
MPV	9.2 ± 1.8	8.8 ± 1.5	9.4 ± 1.9	<0.05	8.8 ± 1.6	9.9 ± 1.9	<0.05^(a)^
Procalcitonin (ng/L)	33.0 (26 – 40.1)	19.1 (11.2 – 28.4)	41.6 (31.3 – 50.9)		22.9 (15.4 – 29.9)	52.9 (39.1 – 66.6)	
Lactate (mmol/L)	4.6 (4.0 – 5.2)	1.9 (1.5 – 2.2)	6.3 (5.5 – 7.0)	<0.05	3.8 (3.0 – 4.2)	6.6 (5.3 – 7.8)	
Albumin (g/dL)	2.6 (2.5 – 2.7)	2.7 (2.6 – 2.9)	2.6 (2.4 – 2.7)	>0.05	2.7 (2.6 – 2.9)	2.5 (2.3 – 2.7)	
BE (mmol/L)	−5.1 (−6.6 – −3.2)	0.1 (−2.8 – 3.7)	−8.1 (−9.5 – −6.7)		−3.7 (−5.5 – −1.1)	−7.7 (−10 – −5.8)	<0.05^(b)^
L/A	1.9 (1.6 – 2.1)	0.7 (0.6 – 0.8)	2.6 (2.3 – 2.9)	<0.05	1.5 (1.2 – 1.7)	2.8 (2.2 – 3.3)	
ICU length (day)	6.8 ± 6.4	7.2 ± 4.2	6.6 ± 7.4		7.4 ± 7.1	5.5 ± 4.2	
Mortality (n(%))	75 (51.4%)	21 (38.2%)	54 (59.3%)	<0.05	47 (47.0%)	28 (60.9%)	<0.05^(c)^

Values are presented as mean ± standard deviation, median (IQR) or number (%), (a) t-test, (b) Mann-Whitney U test, (c) χ^2^ test.

HR: Heart rate, RR: Respiratory rate, MAP: Mean Arterial Pressure, SOFA: Sequential organ failure assessment, WBC: White blood cell, PLT: Platelet count, MPV: Mean Platelet Volume, BE: Base excess, ICU: intensive care unit

### 3.2. Clinical and laboratory characteristics

There were statistically significant differences in mean blood pressure, respiratory rate, SOFA score, procalcitonin, platelets, BE, lactate, L/A ratio, duration of intensive care treatment, and mortality rate between the sepsis and septic shock groups. Similarly, there were also significant differences in lactate, L/A ratio, procalcitonin, BE, duration of intensive care treatment, and mortality rate between the normal platelets group and the decreased platelets group.

**Fig. 1.**
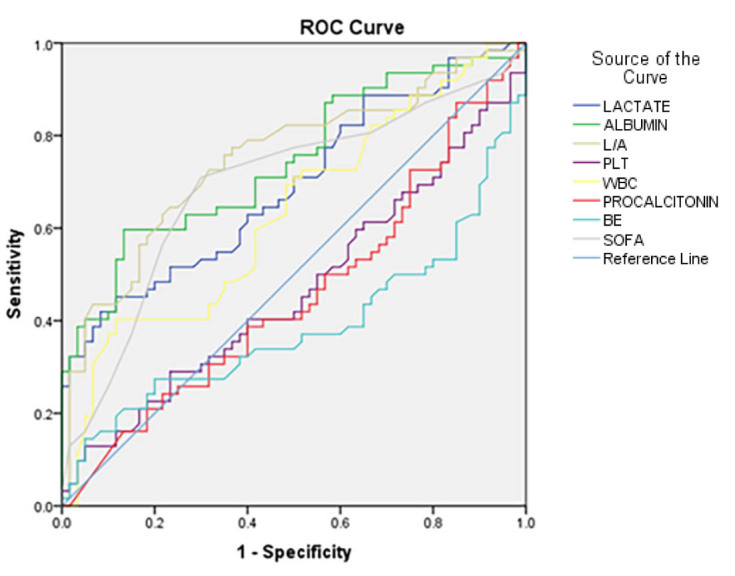
ROC curve of various laboratory parameters in predicting mortality of sepsis patients

**Table 3:** Prognostic values of different parameters in the study

**Values Variables**	AUROC*	**Cut-off threshold**	**Sensitivity (%)**	**Specificity (%)**	**95% CI**
Lactate (mmol/L)	0.71	6.1	44	90.1	0.63 – 0.79
Albumin (g/dL)	0.74	2.7	77.3	60.6	0.66 – 0.82
L/A	0.76	2.1	58.7	88.7	0.68 – 0.83
PLT (G/L)	0.65	101.5	48	87.8	0.55 – 0.76
WBC (G/L)	0.48	#	#	#	0.36 – 0.59
Procalcitonin (ng/L)	0.43	#	#	#	0.32 – 0.55
BE (mmol/L)	0.44	#	#	#	0.32 – 0.55
SOFA	0.70	6.5	72	67.3	0.59 – 0.80

AUROC: Area under the Receiver operating characteristic curve

**Table 4:** Compare L/A to lactate, albumin, procalcitonin, PLT and SOFA score with Delong test

**Values Variables**	**L/A (AUROC = 0.76)**	
	**z-statistic**	**p**
Lactate (AUROC = 0.71)	3.41	0.006
Albumin (AUROC = 0.74)	2.14	0.03
Procalcitonin (AUROC = 0.43)	2.79	0.005
SOFA (AUROC = 0.7)	1.167	0.24
PLT (AUROC = 0.65)	4.67	<0.001
BE (AUROC = 0.44)	2.21	0.02
WBC (AUROC = 0.48)	3.72	<0.001

### 3.3. Prognostic value in sepsis patients

The area under the receiver operating characteristic curve (AUROC) of the L/A ratio in predicting mortality in the study is 0.76. The cutoff point for the L/A ratio at 2.1 showed a sensitivity of 58.7% and specificity of 88.7%. The AUROC of platelets in the study is 0.65. At a cutoff point of 101.5, the sensitivity was 48% and specificity was 87.8%. The prognostic value of the L/A ratio in clinical practice was significantly higher compared to lactate, albumin, procalcitonin, PLT, BE, WBC. The prognostic value of the L/A ratio was also high in elderly, MODS patients.

**Table 5:** Prognostic values of L/A ratio in certain some special groups

**Values Variables**	**AUROC L/A**	**Cut-off threshold**	**Sensitivity (%)**	**Specificity (%)**	**AUROC lactate**	**AUROC albumin**
Septic shock	0.79	2.1	77.8	78.4	0.73	0.77
MODS*	0.78	2.1	66.7	86.3	0.73	0.76
≥ 65 years-old	0.75	2.1	60.0	86.5	0.72	0.72

MODS: Multi-organ dysfunction syndrome

**Table 6:** Multivariate logistic regression analysis of mortality prediction in sepsis patients

**Values Variables**		**OR**	**95% CI**	**p**
Age (years)		1.51	0.666 – 3.562	0.312
Gender	Female	--		0.918
	Male	0.918	0.386 – 2.184	
Infection stage	Sepsis	--		0.038
	Septic shock	3,558	1.071 – 11.817	
SOFA		1.266	1.036 – 1.549	0.021
Platelet		1.005	1.001 – 1.008	0.008
L/A		1.134	1.068 – 1.205	<0.001

CI: Confidence interval; OR: Odd ratio

Multivariate logistic regression analysis demonstrated that the L/A ratio, platelet, SOFA score and septic shock condition were significant predictors of mortality in patients with sepsis (p < 0.05)

## Discussion

Evaluation of clinical characteristics and biomarker values are of great significance when attempting to improve the accuracy of early diagnosis, determining the severity of disease, and predicting the prognosis in sepsis. The ability to diagnose early sepsis and to be able to prognosticate can significantly affect active treatment and subsequently reduce mortality rates. In developing countries, SOFA score, APACHE II, WBC, procalcitonin, CRP, lactate, BE, albumin, and platelets are typically used for prognostication of severity in clinical practice. Thrombocytopenia in sepsis is believed to be related to various mechanisms including disorders in platelet production, dysfunction of platelet receptors, immunological intermediary effects, platelet isolation, and destruction due to coagulation disorders [12]. During sepsis, dysregulated inflammation and coagulation activation lead to excessive platelet activation and consumption, reflecting the severity of systemic inflammation and microvascular dysfunction. This may explain why thrombocytopenia is associated with worse clinical outcomes. We observed that on the first day of admission to the ICU, 31.5% of sepsis and septic shock patients experienced thrombocytopenia with 8.9% of those patients having mild thrombocytopenia, 21.2% having moderate thrombocytopenia, and 1.4% with severe thrombocytopenia. In our study, thrombocytopenia was associated with mortality in patients with sepsis and septic shock and demonstrated modest prognostic performance (AUROC 0.65). As a comparison, Chakradhar Venkata’s et al. study of 304 sepsis and septic shock patients found the prognostic value of thrombocytopenia for mortality with an AUROC was 0.78 [13]. Burunsuzoğlu et al. also reported thrombocytopenia as a risk factor for mortality in patients with sepsis and septic shock in the emergency intensive care setting [14].

Lactate and albumin are two additional parameters that help predict mortality rate and are widely used for early diagnosis, management, and risk stratification in patients with sepsis and septic shock. Elevated lactate levels reflect tissue hypoperfusion and impaired cellular metabolism, while hypoalbuminemia may indicate systemic inflammation, capillary leakage, and poor nutritional status. While these two values can be evaluated independently, our study found that the combination of these two parameters in the lactate/albumin ratio provided a better predictor of prognosis, particularly in critically ill and/or elderly patients. In this study, we found that the lactate/albumin ratio is a significant prognostic indicator in both sepsis and septic shock patients, with an AUROC of 0.76, sensitivity of 58.7%, and specificity of 88.7%. According to Ralphe Bou Chebl's study, the L/A ratio with a cutoff point of 1.47 had an AUROC value 0.66 with a sensitivity of 60% and specificity of 67% [15], Michael Lichtenauer et al. also reported an AUROC 0.7 of L/A ratio for risk Stratification in Sepsis Patients [16]. Similarly, Liu Qiang et al. study on 539 patients indicated an AUROC of 0.743 for L/A, 0.71 for lactate, and 0.659 for albumin [17]. The lactate/albumin (L/A) ratio may better reflect metabolic stress and physiological reserve, potentially explaining its superior prognostic performance compared to either marker alone.

In our study of 146 patients with sepsis and septic shock, we evaluated the prognostic value of several factors including white blood cells, procalcitonin, SOFA score, lactate, albumin, and base excess, which are feasible parameters in some developing countries, with AUROCs of 0.48, 0.43, 0.7, 0.71, 0.74, and 0.44, respectively. Multivariate logistic regression analysis demonstrated that the L/A ratio, platelet, SOFA score and septic shock condition were significant predictors of mortality in sepsis patients. Several studies have also highlighted the value of these parameters in predicting mortality in patients with sepsis and septic shock. Li Fuxing et al. reported AUROCs of L/A 0.65, PCT 0.62, and SOFA score 0.83 [18], Liu Qiang et al. [17] showed AUROCs of L/A 0.743 and SOFA score 0.752, while Kamile Y. et al. reported AUROCs of L/A 0.775 and PCT 0.478 [19]. In elderly patients and patients with multi-organ dysfunction syndrome (MODS), we determined that AUROCs of L/A ratio was higher than lactate, albumin and other prognostic factors. Actually, in critically ill patients, the value of lactate, BE, and platelet count in prognostication was decreased, likely due to confounding factors such as renal failure and/or hepatic failure [20]. In summary, our study suggests that the lactate/albumin ratio has superior prognostic significance. It is easy to implement in developing countries and may more accurately predict mortality in hospitalized patients with sepsis or septic shock.

## Conclusion

The lactate/albumin ratio with a cutoff of 2.1 is an available effective tool for predicting the severity of patients with sepsis and septic shock in developing countries.

## Study limitations

Given the single-center study, the findings should be regarded as hypothesis-generating. Larger multicenter prospective studies incorporating multivariable analyses and diverse patient populations, particularly critically ill and multi-organ failure patients, are needed to clarify the prognostic value of the L/A ratio for mortality in sepsis.

## Appendix

**Table:** SOFA score

**Score**	**0**	**1**	**2**	**3**	**4**
Respiratory system (PaO2/FiO2 [mmHg (kPa)])	> 400 (53.3)	≤ 400 (53.3)	≤ 300 (40)	< 200 (26.7) and mechanically ventilated including CPAP	< 100 (13.3) and mechanically ventilated including CPAP
Coagulation Platelets (×103/μl)	> 150	≤ 150	≤ 100	≤ 50	≤ 20
Liver Bilirubin (mg/dl) [μmol/L]	< 1.2 [< 20]	1.2–1.9 [20–32]	2.0–5.9 [33–101]	6.0–11.9 [102–204]	>12.0 [>204]
Cardiovascular system Mean arterial pressure OR administration of vasopressors required	MAP ≥70 mmHg	MAP< 70 mmHg	dopamine ≤ 5 μg/kg/min or dobutamine (any dose)	dopamine > 5 μg/kg/min OR epinephrine ≤ 0.1 μg/kg/min OR norepinephrine ≤ 0.1 μg/kg/min	dopamine > 15 μg/kg/min OR epinephrine > 0.1 μg/kg/min OR norepinephrine > 0.1 μg/kg/min
Central nervous system Glasgow coma scale	15	13 – 14	10 – 12	6 – 9	< 6
Renal function Creatinine (mg/dl) [μmol/L] (or urine output)	< 1.2 [< 110]	1.2–1.9 [110–170]	2.0–3.4 [171–299]	3.5–4.9 [300–440] (or < 500 ml/day)	> 5.0 [> 440] (or < 200 ml/day
